# Notes and Recollections of a Professional Life

**Published:** 1846-10

**Authors:** 


Art. I.—*Notes and Recollections of a Professional Life.* By the late William Fergusson, Esq., m.d., Inspector-General of Military Hospitals. Edited by his Son, James Fergusson.—*London,* 1846. 8vo, pp. 248.

After having served with distinction for twenty-three years, during the stirring and eventful period between 1794 and 1817, and having attained the rank of Inspector-General of Army Hospitals, Dr. Fergusson retired into civil life and established himself, first at Edinburgh, and, four years afterwards, at Windsor, where he enjoyed an extensive and lucrative practice, till attacked with paralysis two years previous to his death. He possessed ample opportunities during his military career of studying the diseases of soldiers, both on the Continent and in the West Indies, in the field and in garrison, in the time of peace and during war.

"I embarked," he remarks, "nine times from the shores of Britain with armaments on foreign expeditions, and, during twenty-four years' actual service, I spent seventeen years, or parts of them, in other climates, passing through every grade of medical rank in every variety of service, even to that of the sister service of the navy; and it thus was my fortune to have sailed in every ship of war, from the first rate of the line down to the smallest craft that carries a pennant."

Dr. Fergusson contributed many able papers to the medical journals, on subjects connected with the public health, and more particularly on those relating to military hygiene. The latter half of the volume before us consists of a reprint of some of the more important of these contributions, while the first part contains a series of papers, commenced shortly before his illness, and which was intended to form "a digested abstract of opinions and observations that have been subjected to the ordeal of long and deliberate consideration." Unfortunately he was not spared to carry out his views on this point. Of the papers he had completed, some refer to the state of the army as it was when our author served in it, and exposes abuses which happily no longer exist, while others are perhaps too purely military to demand lengthened notice from us. We would recommend the perusal of the work to every army medical officer who feels really interested in the soldier, and takes a pride in the service to which he belongs, as the production of an able and intelligent officer, and containing many excellent observations on subjects connected with the health of troops; it has also the advantage of being written in an amusing and very readable style. We shall content ourselves with one quotation from a paper "On Fever as an Army Disease," which we believe contains the true secret of its successful treatment, and may suggest useful reflections to those who are instant in season and out of season with their endeavours to cut short and to cure it by heroic remedies.

"It is the *gastro-enterite,* cry the disciples of Broussais, and there can be no cure but the leech. It is cerebral inflammation, respond the followers of Clutterbuck, and the only remedy is venesection. Purgatives are the true treatment, proclaims the Edinburgh school. They are irritating and dangerous, replies that of London. In fact, all are equally right and all are equally wrong if they fail to note times and seasons, the nature of the epidemic, and the characteristic tendencies of the patient's constitution, his powers to bear the operation of medicine, and his ability to resist the tendency to death. There can be no treatment of fever by physic but in studying the *juvantia* and *lædentia* of the case—cultivating the first, eschewing the last, and never forgetting that there is a mighty power always operating in your favour—the *vis medicatrix naturæ.* Do not thwart her beyond the mark, and she will get you through difficulties with which, without her aid, you could not cope; but the physician who believes that he possesses beyond these, medicines of specific power in fever, really should have his own licence suspended, and himself put under cure until the monomania subsides. . . . . . . . The battle is to be fought by the nurse, whether in the shape of physician or other attendant it matters not. Only let that attendant be sagacious and diligent, and the patient is saved—the contrary, and he dies."

